# P101: Product dose considerations for real-world hand sanitiser efficacy

**DOI:** 10.1186/2047-2994-2-S1-P101

**Published:** 2013-06-20

**Authors:** J Hines, P Alper, A Voss, A McGeer

**Affiliations:** 1 Research & Development, Deb Group Ltd, Derby, UK; 2 Business Development, Deb Worldwide Healthcare Inc, Brookline, MA, USA; 3 Canisius-Wilhelmina Hospital, Nijmegen, the Netherlands; 4 Mount Sinai Hospital, Toronto, ON, Canada

## Introduction

Alcohol based hand rubs (ABHRs) are extremely effective at reducing microbial contamination and are central to established best practices for hand hygiene. Modern dispensing systems have brought benefits such as hygienically sealed cartridges with integral pumps for dosing liquids, gels or foams. A remaining issue concerns the measured efficacy of such products and their use in practice as dictated by pump volume.

## Objectives

ABHRs for professional use must demonstrate efficacy through standard in-vivo tests; either EN1500 or ASTM E1174. Products typically pass such tests using standard doses not necessarily related to real use by healthcare workers. In this study we set out to identify the optimal dose for an ABHR product based on observation of real behavior.

## Methods

Data from the DebMed GMS Hand Hygiene Monitoring System was used to establish product dose applied in clinical settings via the number of dispenser presses per hand hygiene event. In a separate study healthcare workers applied ABHR using best-practice methodology to establish product drying time as a function of dose. Hand coverage was assessed using a laboratory method based on product coverage. In-vivo efficacy was established using both ASTM E1174 and EN 1500 methods.

## Results

Healthcare workers intuitively calibrate ABHR dose based on drying time, hand coverage and product ergonomics. In our studies using Deb dispensers, over 90% of healthcare workers used a single pump of ABHR, even when multiple pumps are indicated. Our studies established that 1.5ml of ABHR in foam format dries in approximately 30 seconds and fully covers most hands. Both ASTM 1174 and EN 1500 tests confirmed that 1.5ml of Deb ABHR in foam format with 30 seconds contact time was sufficient to provide effective hand hygiene.

## Conclusion

Product drying time, hand coverage and measured efficacy data are considered to determine the optimal dose for an ABHR in foam format dispensed from a sealed cartridge wall-mounted unit. This is combined with observed behaviour of healthcare workers to design a pump that delivers the optimal dose in a single press, connecting for the first time measured in-vivo efficacy standards with real-world use to ensure effective sanitization.

## Disclosure of interest

None declared

